# Preventive Effect of Bifidobacterium Supplementation on Neonatal Cholestasis in Preterm Neonates with Very Low Birth Weight

**DOI:** 10.1155/2020/4625315

**Published:** 2020-03-10

**Authors:** Gaohong Wu, Xiaoqian Chen, Ningxun Cui, Yunxia He, Jiaying Fan, Dongya Yan, Xueping Zhu, Xiaoli Zhu

**Affiliations:** ^1^Department of Neonatology, Children's Hospital of Soochow University, Suzhou, Jiangsu 215025, China; ^2^Department of Intervention, The First Affiliated Hospital of Soochow University, Suzhou, Jiangsu 215006, China

## Abstract

**Background:**

Cholestasis is a common but serious clinical condition in preterm neonates. The current management for preterm neonatal cholestasis has limitations. The aim of this study was to determine effects of Bifidobacterium supplementation on the prevention and alleviation of cholestasis in preterm infants with very low birth weight.

**Methods:**

Preterm neonates with very low birth weight were enrolled in the Children's Hospital of Soochow University between December 2012 and December 2017. The patients were randomly assigned into Bifidobacterium and control groups, and effects of Bifidobacterium supplementation on the outcomes were compared between the two groups.

**Results:**

There was no significant difference in the baseline characteristics in the two groups. Notably, the proportion of cases with neonatal cholestasis was significantly lower, with fewer neonatal cholestasis-associated complications in the Bifidobacterium group compared with the control group (6% versus 22%, *P* < 0.01). Furthermore, the Bifidobacterium group exhibited less severe cholestasis and better improvement of the liver function than the control group as evidenced by the biochemical tests (*P* < 0.01). Furthermore, the Bifidobacterium group exhibited less severe cholestasis and better improvement of the liver function than the control group as evidenced by the biochemical tests (*P* < 0.01). Furthermore, the Bifidobacterium group exhibited less severe cholestasis and better improvement of the liver function than the control group as evidenced by the biochemical tests (*days*, *P* < 0.01). Furthermore, the Bifidobacterium group exhibited less severe cholestasis and better improvement of the liver function than the control group as evidenced by the biochemical tests (*P* < 0.01). Furthermore, the Bifidobacterium group exhibited less severe cholestasis and better improvement of the liver function than the control group as evidenced by the biochemical tests (*P* < 0.01). Furthermore, the Bifidobacterium group exhibited less severe cholestasis and better improvement of the liver function than the control group as evidenced by the biochemical tests (*P* < 0.01). Furthermore, the Bifidobacterium group exhibited less severe cholestasis and better improvement of the liver function than the control group as evidenced by the biochemical tests (*P* < 0.01). Furthermore, the Bifidobacterium group exhibited less severe cholestasis and better improvement of the liver function than the control group as evidenced by the biochemical tests (

**Conclusions:**

Bifidobacterium supplementation has significantly preventive and other beneficial effects on the management of cholestasis in preterm infants with very low birth weight. Its long-term safety and effectiveness will need further investigation. This trial is registered with the Chinese Clinical Trial Registry (Registration No. ChiCTR1900022296).

## 1. Background

Cholestasis commonly occurs in preterm neonates and is attributed to multiple causative factors (e.g., abnormality of the gastrointestinal and liver tracts, infection, immaturity of red blood cells, and genetic metabolic diseases) [1]. Preterm cholestasis is worsened by the delay in the enteral feeding, mainly because it increases enterohepatic circulation and elevates levels of serum bilirubin [2, 3]. The main characteristics of neonatal cholestasis include the pathological jaundice with an accumulation of bilirubin in the blood, an enlargement of the liver, darkening of the feces color, and abnormally higher levels of serum alanine aminotransferase (ALT). Previous laboratory examinations in neonatal cholestasis have shown that the levels of serum ALT and/or aspartate aminotransferase (AST) markedly increased, and total bilirubin (TB) < 85 *μ*mol/L (5 mg/dL), conjugated bilirubin > 17.1 *μ*mol/L (1 mg/dL) or serum TB > 85 *μ*mol/L, conjugated bilirubin/total bilirubin > 20% in neonates with cholestasis [4–6].

The current recommendations for the management of the preterm neonatal cholestasis, such as conservative phototherapy and surgical intervention, have limitations, and there are a number of concerns over adverse effects. Therefore, prevention and alternative treatments would be valuable in this regard. In addition, there is a need for an improvement and better care for cholestasis in preterm neonates and those with cholestasis. Notably, the gastrointestinal function in newborns especially preterm infants is still immature, for which parenteral nutrition (PN) is required. The implementation of PN and delay in the enteral feeding may cause PN-associated cholestasis (PNAC) or even make the existing cholestasis worse. Long-term PN can also result in an imbalance in the intestinal flora, damage of the gastrointestinal mucous, and disruption in the bile secretion and further aggravate the conditions of cholestasis [7]. Therefore, it is important to correct the imbalanced intestinal flora by increasing colonization of beneficial bacteria and to improve the function of the gastrointestinal function in preterm infants. In fact, a number of clinical trials have reported that preterm infants with prebiotics had improvement in gastrointestinal motility, stool characteristics, and enteral feeding tolerance [8–10]. Moreover, prebiotics had impact on the growth of beneficial bacteria in the gut [11, 12], alleviation of the cell damage in the small intestine, reduction of intestinal inflammation, promotion of maturation of the intestinal function, and improvement of the stool frequency and shape, as well as enteral feeding tolerance in preterm infants [13]. However, it remains to be further investigated for the effect of prebiotic supplementation on the prevention and severity of neonatal cholestasis in preterm neonate with very low birth weight.

In this study, we aimed to assess effects of prebiotic supplementation on the potential prevention and alleviation of severity of neonatal cholestasis in preterm neonate with very low birth weight. The findings obtained through conducting this study may provide scientific evidence of the prebiotic supplementation in the better management of preterm newborns with very low birth weight.

## 2. Methods

### 2.1. Human Subjects and Study Design

In this prospective, double-blinded, randomized comparative study, 510 preterm neonates in the Children's Hospital of Soochow University between December 2012 and December 2017 were screened for their eligibility. The following inclusion criteria were used: (1) admission to the hospital within 12 hours after birth, (2) gestational ages ranging from 28 to 34 weeks, (3) very low birth weight (<1500 g), and (4) total parenteral nutrition (TPN) one day following birth. However, the patients who presented the following conditions were excluded from this study: (1) congenital heart diseases, malformations in the gastrointestinal (GI) tract, and genetic metabolic diseases; (2) surgical treatment during hospitalization; (3) severe symptoms of digestive diseases (e.g., vomiting, abdominal distention, and diarrhea) before TPN; (4) cholestasis at the time of admission to our hospital; (5) taking gastrointestinal stimulants; (6) taking antibiotics; and (7) incompletion or withdrawal of treatment during hospitalization. Ten patients were excluded from this study, including one congenital birth defect, five severe symptoms of digestive diseases before TPN, and four cholestasis diagnosed at the time of admission. As a result, a total of 500 study subjects were enrolled in this study.

All the guardians/parents of the infants had given written informed consent prior to enrollment in the study. This study was reviewed and approved by the Ethics Committee of the Children's Hospital of Soochow University (Suzhou, Jiangsu, China).

### 2.2. Treatment with Bifidobacterium Supplementation

All the study preterm neonates were randomly assigned into the Bifidobacterium group and the control group using the stratified permuted block randomization. The patients in the Bifidobacterium group were given orally Bifidobacterium triple viable powder (Shanghai Xinyi Pharmaceutical Company, Peifeikang powder, 0.5 g/time, 3 times/day) within 24 hours after birth until TPN was implemented, while those neonates in the control group were orally fed without Bifidobacterium supplementation. Bifidobacterium triple viable powder contains live bacteria of long Bifidobacterium, *Lactobacillus acidophilus*, and *Enterococcus faecalis* at no less than 1.0 × 10^7^ cfu/g [14, 15].

Before having reached enteral feeding, PN was implemented to prevent hypoglycemia, hyperglycemia, and electrolyte acid-base balance disorders. In addition to routine hepatoprotective therapy (including intravenous drip of energy mixture, fat-soluble vitamin K, and other symptomatic treatments), ursodeoxycholic acid (produced by German Hooker Pharmaceutical Factory, 250 mg/grain, 10-15 mg/kg·d, orally, twice) combined with jaundice Yinchen granules (Shanghai Jing'an Pharmaceutical Company, 20 g/package, 3 g/time, orally, three times a day) was used for the treatment of cholestasis.

Both groups were fed with formula milk powder designed for preterm infants (osmotic pressure 327 mOsm/L) with an initial 3-5 mL/(kg·d), less than 20 mL/(kg·d). The daily milk quantity and calorie of each infant were monitored to determine at least 90-120 kcal/(kg·d) [16].

The formula of PN includes 20% medium-long chain fat emulsion, water-soluble vitamins, fat-soluble vitamins, amino acids essential for infants, glucose, electrolytes, and trace elements. The initial dosage of amino acid was 1.5-2.0 g/(kg·d), then gradually increased to 3.5-4.0 g/(kg·d); the initial dosage of fat milk was 0.5-2.0 g/(kg·d), which gradually increased to 2.0-3.0 g/(kg·d); and the dosage of glucose gradually increased from 4-6 mg/(kg·min) to 11-14 mg/(kg·min). PN was administered through a peripheral venous micropump for 8-12 hours.

### 2.3. Biochemical Examinations and Relevant Diagnostic Criteria

The levels of TB, direct bilirubin (DB), gamma glutamine transferase, total bile acid (TBA), and total cholesterol (TC) in the infant patients with cholestasis were recorded. The diagnosis of cholestasis was primarily made according to the following biochemical examinations: TB < 85 *μ*mol/L (5 mg/dL), conjugated bilirubin > 17:1 *μ*mol/L (1 mg/dL) or serum TB > 85 *μ*mol/L, and conjugated bilirubin/total bilirubin > 20%. Biochemical tests for the liver function were performed every 5 days in the study subjects. Cholestatic liver damage was diagnosed based on the results of blood tests indicating that the serum bilirubin, alkaline phosphatase, gamma glutamine transferase, ALT, and AST level were increased when the child experienced jaundice, hepatomegaly, anorexia, dark urine color, light stool color, and other clinical symptoms. Necrotizing enterocolitis (NEC) in newborns was diagnosed based on the clinical presentation of symptoms such as bloody stool, abdominal distention, vomiting, lethargy, apnea, and hypotonia and if abdominal X-ray examination showed cystic emphysema of the intestinal wall. However, atypical clinical manifestations required differentiation from other diseases. Extrauterine growth retardation (EUGR) was diagnosed if the weight, length, or head circumference of the child was below the 10th percentile of the expected value for the corresponding intrauterine growth rate at the time of discharge. The clinical manifestations of septicemia were nonspecific, mainly including poor response, fever or lack of temperature rise, lack of weight increase or slow growth, jaundice, hepatosplenomegaly, shock, and even multiple organ lesions. The results of nonspecific examination included increased or decreased peripheral blood leukocyte count, a ratio of baculonuclear cells to neutrophils of ≥0.16, a platelet count of <100 × 109/L, increased C-reactive protein level, increased serum procalcitonin level, and increased IL-6 level. Septicemia was diagnosed based on clinical manifestations and confirmation of any of the following conditions: (1) pathogenic bacteria identified in blood culture or sterile coelomic fluid culture and (2) if opportunistic pathogens were found in blood culture, the same bacteria were also found in another blood culture, catheter tip, or sterile body cavity. Presentation of clinical manifestations and any of the following conditions resulted in a clinical diagnosis of septicemia: (1) at least two abnormal nonspecific examination results and (2) positive result on pathogen antigen or DNA testing. All diagnostic criteria were obtained from literature reports of studies conducted outside of China [11–13, 17].

### 2.4. Statistical Analysis

SPSS17.0 statistical software was used for the statistical analysis in this study. The data with normal distribution were expressed as the mean ± standard deviation (SD), and the independent sample *t*-test was used to compare the means of two sets of data. For data without satisfying the normal distribution, they were expressed as the median value ± interquartile range (IQR), and the two groups were compared by a nonparametric rank sum test that analysed the difference. The Chi-squared (*χ*^2^) test was used for comparison of enumeration data of two paired groups. *P* < 0.05 was considered as significant difference in all statistical tests.

## 3. Results

### 3.1. Baseline Characteristics of the Study Subjects

A total of 511 preterm neonates were evaluated for eligibility, of which 500 infants were finally enrolled in this study. The study patients were randomly assigned into two groups with 250 neonates in the control group and in the Bifidobacterium supplementation group. The baseline demographic and clinical characteristics of the study patients, including methods of childbirth, Apgar scores, gestational age, and gender, were summarized in Suppl. [Supplementary-material supplementary-material-1]. There was no significant difference in the baseline characteristics in the two study groups (Suppl. [Supplementary-material supplementary-material-1]). In addition, no cholestasis was identified in the two groups prior to an intervention.

### 3.2. Comparison of Incidence Rates of Neonatal Cholestasis and Its Associated Complications in the Study Subjects with or without Bifidobacterium Supplementation

To determine a potentially preventive effect of Bifidobacterium supplementation on neonatal cholestasis, we compared incidence rates of neonatal cholestasis and its associated complications in the two groups with or without Bifidobacterium supplementation. As presented in Table 1, a proportion of cases with neonatal cholestasis were significantly lower in the Bifidobacterium group in contrast to the control group (6% versus 22%, *P* < 0.01). Additional analysis of neonatal cholestasis-associated complications showed that the Bifidobacterium group exhibited significantly lower proportion of cases that had the following cholestasis-associated complications compared with the control group: cholestatic liver injury (6.67% versus 36.36%, *P* < 0.05), NEC (0.8% versus 4.8%, *P* < 0.01), FI (6% versus 12%, *P* < 0.01), and EUGR (8.4% versus 14.8%). It was of note that a proportion of septicemia was slightly lower in the neonates with Bifidobacterium supplementation compared with those with placebo, whereas the difference between the two groups was not statistically different (1.2% versus 1.6%, *P* = 0.725).

### 3.3. Comparison of the Severity of Neonatal Cholestasis in the Study Subjects with or without Bifidobacterium Supplementation

We also examined effect of Bifidobacterium supplementation on the severity of neonatal cholestasis in the study infants, which were assessed on the basis of a range of biochemical tests for the disease severity. As shown in Table 2, neonatal cholestasis was significantly less severe in the Bifidobacterium group than the control group, as evidenced by significantly lower levels of peak total bilirubin (168 ± 76 versus 230 ± 160 *μ*mol/L, *P* < 0.05), peak direct bilirubin (73 ± 19 versus 86 ± 21 *μ*mol/L, *P* < 0.05), *γ*-glutamine transferase (219 ± 60 versus 285 ± 70 U/L, *P* < 0.05), alkaline phosphatase (322 ± 50 versus 513 ± 100 U/L, *P* < 0.05), total bile acid (69.0 ± 10.0 versus 74.0 ± 11.0 *μ*mol/L, *P* < 0.05), and total cholesterol (2.0 ± 1.3 versus 2.6 ± 0.8 mmol/L, *P* < 0.05).

### 3.4. Comparison of the Liver Function in the Study Subjects with or without Bifidobacterium Supplementation

We also compared effect of Bifidobacterium supplementation on the improvement of the liver function in the study infants based upon the laboratory tests for the liver function. As detailed in Table 3, the liver function was significantly improved in the Bifidobacterium group versus the control group, as evidenced by the laboratory test results for the measurement of the liver function (*P* < 0.05).

### 3.5. Comparison of Duration of Hospitalization and Other Clinical Outcomes in the Study Subjects with or without Bifidobacterium Supplementation

Finally, we made comparison of the duration of hospitalization and other clinical outcomes between the two groups with or without Bifidobacterium supplementation, and the main findings were summarized in Table 4. Duration of hospitalization was significantly shorter in the Bifidobacterium group than those in the control group (14.45 ± 2.13 versus 16.12 ± 2.22 days, *P* < 0.01). The Bifidobacterium group exhibited significantly better clinical outcomes in comparison with those in the control group (*P* < 0.05) over the period of this study as evidenced as follows: days required for the study neonates to meet the full enteral feeding were significantly fewer in the Bifidobacterium group than those in the control group (9.2 ± 2.11 versus 12 ± 5.67 days, *P* < 0.01), duration of meconium passage was significantly shorter in the Bifidobacterium group than that in the control group (5.0 ± 3.6 versus 6.6 ± 3.38 days, *P* < 0.05), proportion of cases on fasting and duration of fasting were significantly lower in the Bifidobacterium group than those in the control group (0.8% versus 5.6%, *P* < 0.05; 3.0 ± 1.6 versus 5.6 ± 2.38 days, *P* < 0.01, respectively), and duration of weight gain to normal was significantly shorter in the Bifidobacterium group than those in the control group (4.77 ± 2.49 versus 6.87 ± 2.71 days, *P* < 0.01).

## 4. Discussion

This study of effects of Bifidobacterium supplementation on cholestasis in preterm infants with very low birth weight has the following main novel findings: (1) Bifidobacterium supplementation significantly reduced the risk for neonatal cholestasis and its related complications in the preterm infants with very low birth weight (Table 1); (2) Bifidobacterium supplementation was significantly associated with less severity of neonatal cholestasis and better improvement of the liver function in the preterm infants with very low birth weight (Tables 2 and 3); and (3) Bifidobacterium supplementation significantly shortened the time to reach the full enteral feeding, duration of hospitalization, meconium passage, and duration of weight gain to normal and improved other outcomes in the preterm infants with very low birth weight (Table 4). These findings suggested that Bifidobacterium supplementation has significantly preventive and other beneficial impacts on the management of cholestasis in preterm infants with very low birth weight.

To our knowledge, the studies of efficacy of Bifidobacterium administration on cholestasis in the preterm infants with very low birth weight are limited. Many previous studies focused on evaluation of impacts of prebiotics on the care for preterm neonates, and benefits of prebiotic administration included improvement of the stool characteristics, reduction of the enteral feeding tolerance, and increase in the gastrointestinal motility [8–10, 18–22]. Wang and colleagues reported that the incidence rates of cholestasis in preterm neonates with oral administration of Bifidobacterium supplementation had significant lower incidence rates of cholestasis [21]. Another study showed that early administration of Bifidobacterium reduced the risk for the development of cholestasis, improved the feeding intolerance, and promoted the excretion of meconium in preterm infants [23]. However, the challenges in the management of the preterm infants and prevention of cholestasis, in particular, those with very low birth weight, still remain [24]. In the present study, the infants were preterm with very low birth weight (ages of gestation between 28 and 34 weeks, weighting fewer than 1500 g) in accordance with the World Health Organization (WHO) criteria, with extremely preterm (<28 weeks), very preterm (28-32 weeks), and moderate to late preterm (32-37 weeks).

Bifidobacterium has been detected in fecal samples of infants at the ages of 3–276 days and is among the main beneficial bacteria with a minimum concentration of 1.0 × 10^7^ cfu/g [15]. According to the instructions provided by the manufacturer, Bifidobacterium supplementation is used in various dosages/schedules, and the dose for children aged 1–5 years is 1.0 g/time given 3 times/day. Considering that the number of Bifidobacterium in the human body decreases with age, we lowered the dose to 0.5 g/time, 3 times/day for the newborns in the current study, which was half of the dose for children. Notably, the clinical outcomes of the infants were satisfactory in our study. We found that Bifidobacterium supplementation improves enteral feeding tolerance, time to reach the total enteral feeding, and other good clinical outcomes in preterm infants with very low birth weight. It was likely that Bifidobacterium led to increase in the gastrointestinal motility and gastric emptying through stimulating the secretion of motilin. More rapid gastric emptying and reduction of gastric residues may result in improvement of enteral feeding tolerance. Moreover, probably owing to these beneficial effects of Bifidobacterium supplementation, both total serum bilirubin and peak bilirubin levels were reduced by the use of Bifidobacterium supplementation in our study. The present study, together with those of others, showed that the enteral feeding tolerance was improved with the administration of Bifidobacterium supplementation [25]. We also found other beneficial effects of Bifidobacterium supplementation in the preterm infants with very low birth weight, lowering of peak bilirubin and more rapidly reaching the full enteral feeding.

Our study may have a few potential limitations. First, the duration of intervention was relatively not long, for which the long-term safety and efficacy of Bifidobacterium supplementation on the preterm infants with very low birth weight are unknown. Second, Bifidobacterium trifecta contains small amounts of *Lactobacillus acidophilus* and *Enterococcus faecalis*. Although these probiotics have been reported to have little impact on human health [26], it is necessary to understand their effect on cholestasis through one-way analysis of variance. Third, we did not examine the fecal microbiota for bacteria spices, and their association with metabolizing bilirubin remains to be investigated. Fifth, despite the apparently preventive and other beneficial impacts of Bifidobacterium supplementation in the preterm infants with very low birth weight, the underlying mechanisms will require further in-depth studies.

## 5. Conclusions

Taken together, we have found the beneficial effects of Bifidobacterium supplementation for the management of cholestasis in the preterm newborns with very low birth weight, possibly through improving the colonization of the beneficial intestinal bacteria and the enteral feeding tolerance, as well as decreasing the enterohepatic circulation of bilirubin. The long-term safety and effectiveness of Bifidobacterium supplementation will need further clinical investigation with longer duration of intervention in a large sample size.

## Figures and Tables

**Table 1:** Incidence rates of neonatal cholestasis and its associated complications in the study subjects with or without Bifidobacterium supplementation.

	Bifidobacterium group	Control group	*χ* ^2^	*P*
Neonatal cholestasis (%)	15 (6.00)	55 (22.00)	26.578	<0.01
Cholestatic liver injury (%)	1 (6.67)	20 (36.36)	4.948	0.029
Septicemia (%)	3 (1.2)	4 (1.6)	0.145	0.725
NEC (%)	2 (0.8)	12 (4.8)	7.349	<0.01
FI (%)	15 (6.0)	30 (12.0)	17.104	<0.01
EUGR (%)	21 (8.4)	37 (14.8)	4.993	0.035

Note: NEC: necrotizing enterocolitis of newborn; FI: feeding intolerance; EUGR: extrauterine growth retardation. The Chi-squared (*χ*^2^) test was used for comparison of enumeration data of two paired groups. *P* < 0.05 was considered as significant difference between the two groups.

**Table 2:** Biochemical examinations for neonatal cholestasis in the study subjects with or without Bifidobacterium supplementation.

	Bifidobacterium group	Control group	*t*-test	*P*
Peak total bilirubin (*μ*mol/L)	168 ± 76	230 ± 160	2.268	0.026
Peak direct bilirubin (*μ*mol/L)	73 ± 19	86 ± 21	2.975	<0.01
*Γ*-Glutamine transferase (U/L)	219 ± 60	285 ± 70	4.639	<0.01
Alkaline phosphatase (U/L)	322 ± 50	513 ± 100	11.071	<0.01
Total bile acid (*μ*mol/L)	69.0 ± 10.0	74.0 ± 11.0	2.18	<0.05
Total cholesterol (mmol/L)	2.0 ± 1.3	2.6 ± 0.8	2.547	0.013

Note: the independent sample *t*-test was used to compare the means of two sets of data. *P* < 0.05 was considered as significant difference between the two groups.

**Table 3:** Comparative analysis of the laboratory tests for the liver function at different time points between the two groups.

	Bifidobacterium group ($\bar{X}\pm s$)	Control group ($\bar{X}\pm s$)	*t*-test	*P*
Peak total bilirubin (*μ*mol/L)				
Baseline	200 ± 80	260 ± 165	2.121	<0.05
5 days	190 ± 80	250 ± 160	2.174	<0.05
10 days	180 ± 80	235 ± 150	2.097	<0.05
Discharge from hospital	90 ± 20	100 ± 15	2.592	<0.05
Peak direct bilirubin (*μ*mol/L)				
Baseline	93 ± 19	96 ± 21	0.687	<0.05
5 days	85 ± 19	95 ± 20	2.349	<0.05
10 days	80 ± 19	90 ± 20	2.349	<0.05
Discharge from hospital	20 ± 6	25 ± 5	4.149	<0.01
*Γ*-Glutamine transferase (U/L)				
Baseline	220 ± 61	290 ± 71	4.846	<0.05
5 days	200 ± 55	280 ± 71	5.773	<0.01
10 days	190 ± 52	275 ± 68	6.435	<0.01
Discharge from hospital	50 ± 2	60 ± 3	17.97	<0.01
Alkaline phosphatase (U/L)				
Baseline	350 ± 50	520 ± 99	9.934	<0.01
5 days	340 ± 50	500 ± 100	3.851	<0.01
10 days	320 ± 50	490 ± 99	9.934	<0.01
Discharge from hospital	310 ± 50	480 ± 90	10.70	<0.01
Alkaline phosphatase (U/L)				
Baseline	80 ± 40	100 ± 50	2.024	<0.05
5 days after admission	78 ± 40	98 ± 51	2.000	<0.05
10 days	70 ± 40	95 ± 50	2.530	<0.05
Discharge from hospital	68 ± 35	90 ± 45	2.500	<0.05
Total cholesterol (mmol/L)				
Baseline	3.5 ± 1.2	5.0 ± 1.1	5.972	<0.01
5 days	3.3 ± 1.2	4.9 ± 1.0	6.638	<0.01
10 days	3.2 ± 1.1	4.5 ± 0.9	5.928	<0.01
Discharge from hospital	3.0 ± 1.0	4.0 ± 0.9	4.817	<0.01

Note: the independent sample *t*-test was used to compare the means of two sets of data. *P* < 0.05 was considered as significant difference between the two groups.

**Table 4:** Duration of hospitalization and other clinical outcomes in the study subjects with or without Bifidobacterium supplementation.

	Bifidobacterium group	Control group	*t*/*χ*^2^	*P*
Time to reach total enteral feeding (days)	9.2 ± 2.11	12 ± 5.67	*t* = 3.000	<0.01
Duration of meconium passage (days)	5.0 ± 3.6	6.6 ± 3.38	*t* = 2.100	0.039
Percentage of cases on fasting (%)	2 (0.80)	14 (5.60)	*χ* ^2^ = 9.298	0.04
Duration of fasting (d)	3.0 ± 1.6	5.6 ± 2.38	*t* = 5.876	<0.01
Duration of weight gain to normal (days)	4.77 ± 2.49	6.87 ± 2.71	*t* = 3.698	<0.01
Duration of hospitalization (days)	14.45 ± 2.13	16.12 ± 2.22	*t* = 3.518	<0.01

Note: the Chi-squared (*χ*^2^) test was used for comparison of enumeration data of two paired groups. The independent sample *t*-test was used to compare the means of two sets of data. *P* < 0.05 was considered as significant difference between the two groups.

## Data Availability

The datasets used and/or analysed during the current study are available from the corresponding author on reasonable request.

## Abbreviations

ALT: Alanine aminotransferase
AST: Aspartate aminotransferase
TB: Total bilirubin
PN: Parenteral nutrition
PNAC: PN-associated cholestasis
TPN: Total parenteral nutrition
GI: Gastrointestinal
NEC: Enterocolitis of newborn
EUGR: Extrauterine growth retardation
SD: Standard deviation
IQR: Quartile interval.

## Supplementary Materials

Supplementary Materials Suppl. Table 1: baseline characteristics of the study subjects.
