# Supplementary material for: Preventive Effect of Bifidobacterium Supplementation on Neonatal Cholestasis in Preterm Neonates with Very Low Birth Weight
Source: Gastroenterol Res Pract. 2020 Mar 10;2020:4625315. doi: 10.1155/2020/4625315 (PMC7085826; doi:10.1155/2020/4625315)
Supplement: Supplementary Materials — Suppl. Table 1: baseline characteristics of the study subjects. [file 4625315.f1.docx]

Supplementary Materials

**Suppl. Table 1** **Baseline characteristics of the study subjects**

| Characteristics | Bifidobacterium group | Control  group | t/χ^2^ | *P* |
| --- | --- | --- | --- | --- |
| Methods of childbirth |  |  |  |  |
| Cesarean section (%) | 112(45.00) | 106(42.57) | χ^2^=0.293 | 0.652 |
| Natural childbirth (%) | 138(55.00) | 144(56.43) |  |  |
| Apgar scores |  |  |  |  |
| 1 min | 8.90±1.10 | 8.80±0.98 | t=0.440 | 0.661 |
| 5 min | 9.49±0.41 | 9.45±0.49 | t=0.406 | 0.686 |
| Gestational age (weeks) | 32.0±2.6 | 31.3±2.8 | t=1.187 | 0.239 |
| Small for gestational age  (%) | 100(40.00) | 110(44.00) | x^2^=0.821 | 0.415 |
| Appropriate for gestational age (%) | 150(60.00) | 140(56.00) |  |  |
| Gender |  |  |  |  |
| Male (%) | 130 (52.00) | 127 (51.00) | χ^2^=0.072 | 0.858 |
| Female (%) | 120 (48.00) | 123 (49.00) |  |  |
| Birth weight  (grams) | 1240.50±180.55 | 1235.30±163.60 | t=0.138 | 0.890 |
| Age (days) with initial PN | 5.00±3.00 | 6.00±3.00 | t=1.527 | 0.13 |
| PN duration (days) | 9.00±7.00 | 30.0±28.0 | t=4.715 | <0.01 |

**Note:** The Apgar scoring system was used to evaluate the study newborns in the first one minute and five minutes of their lives after they were born. A preterm birth was defined as gestational ages between 28 and 34 weeks in this study. PN: parenteral nutrition. The independent samples t test was used to compare the means of two sets of data. Chi-Square (χ^2^) test was used for comparison of enumeration data of two paired groups. P < 0.05 was considered as significant difference between the two groups.
